# Severe Psychosis, Drug Dependence, and Hepatitis C Related to* Slamming* Mephedrone

**DOI:** 10.1155/2016/8379562

**Published:** 2016-05-10

**Authors:** Helen Dolengevich-Segal, Beatriz Rodríguez-Salgado, Jorge Gómez-Arnau, Daniel Sánchez-Mateos

**Affiliations:** ^1^Dual Pathology Program, Hospital Universitario del Henares, Fundación Psiformación, Avenida Marie Curie, s/n, 28822 Madrid, Spain; ^2^Mental Health Center of San Blas, Hospital Universitario Ramón y Cajal, Madrid, Spain; ^3^Hospital Universitario del Henares, Coslada, 28822 Madrid, Spain; ^4^Hospital Universitario La Fe, Valencia, Spain

## Abstract

*Background*. Synthetic cathinones (SCs), also known as “bath salts,” are *β*-ketone amphetamine compounds derived from cathinone, a psychoactive substance found in* Catha edulis*. Mephedrone is the most representative SC.* Slamming* is the term used for the intravenous injection of these substances in the context of* chemsex* parties, in order to enhance sex experiences. Using IV mephedrone may lead to diverse medical and psychiatric complications like psychosis, aggressive behavior, and suicide ideation.* Case*. We report the case of a 25-year-old man admitted into a psychiatric unit, presenting with psychotic symptoms after* slamming* mephedrone almost every weekend for the last 4 months. He presents paranoid delusions, intense anxiety, and visual and kinesthetic hallucinations. He also shows intense craving, compulsive drug use, general malaise, and weakness. After four weeks of admission and antipsychotic treatment, delusions completely disappear. The patient is reinfected with hepatitis C.* Discussion*. Psychiatric and medical conditions related to* chemsex* and* slamming* have been reported in several European cities, but not in Spain. Psychotic symptoms have been associated with mephedrone and other SCs' consumption, with the IV route being prone to produce more severe symptomatology and addictive conducts. In the case we report, paranoid psychosis, addiction, and medical complications are described.

## 1. Introduction

Synthetic cathinones (SCs) are a group of *β*-ketone amphetamine compounds derived from cathinone, a naturally occurring psychoactive component found in* Catha edulis*, a plant traditionally consumed in the Horn of Africa [1]. SCs belong to the so-called New Psychoactive Substances (NPS) and have been one of the main active components of “bath salts” or “plant feeders.” They are perhaps one of the psychoactive substances most involved in the “legal high” phenomenon.

Initially, they were a legal alternative to other illegal drugs, such as 3,4-methylenedioxy-methamphetamine (MDMA or* ecstacy*), cocaine, or amphetamine, because of their psychoactive properties, like an entactogenic effect similar to the one of MDMA and a stimulating effect like that of cocaine [2].

Among this group of substances, the most popular has been mephedrone, or 4-methylmethcathinone [3], followed by butylone, methylone, and some phenylpyrrolidines, such as methylenedioxypyrovalerone (MDPV) and *α*-pyrrolidinopentiophenone (*α*-PVP) [4]. The control of mephedrone in Europe took place in April 2010 (UK), and it was not until July 2012 that mephedrone and methylone were classified in Schedule I of the Controlled Substances Act in the United States [5, 6].

Several side effects have been widely described as the result of mephedrone consumption, such as cardiovascular disorders, rhabdomyolysis, renal failure, agitation, bizarre behaviors, aggression, and even death. In addition, the addictive potential of mephedrone has been widely described [7]. Its consumption has been associated with increased risk of presenting psychotic symptomatology (delusive thoughts, hallucinations, or disorganized speech) [8]. Although, in most cases, psychosis resolved within a few days, there have been reports of persistent psychotic symptoms for weeks after a single consumption of SCs [9].

Mephedrone has been very popular among young men who frequent nightclubs, especially in men who have sex with men (MSM). Thus, in the UK, it became the sixth most consumed substance in gay bars and nightclubs, after alcohol, tobacco, cannabis, MDMA, and cocaine [10–12]. Recent concern has arisen around the intravenous (IV) ministration of mephedrone and other cathinones in some groups of drug users, especially in MSM who engage in a practice known as* slamming*. This term refers to the IV use of mephedrone and other substances at* chemsex parties*, where subjects frequently exchange syringes and engage in risky sexual behavior [13, 14]. This behavior may lead to medical and psychiatric problems associated with IV drug injection, including the contraction of sexually transmitted diseases (STDs) or viral infections, such as human immunodeficiency virus (HIV) and hepatitis C virus (HCV) [15].

Here, we describe the case of a patient who developed psychotic symptoms after months of* slamming* mephedrone. To our knowledge, this is the first description of psychotic symptoms following the use of mephedrone in the context of* chemsex*.

## 2. Clinical Case

The patient, a 25-year-old male, attends the dual diagnosis clinic of our hospital. He asks for help to stop using drugs, specifically mephedrone taken intravenously, started four months ago. He has a poor physical appearance and admits intense weight loss and poor self-care. He is offered hospital admission but rejects it, so a medical appointment is scheduled a few days after. He does not attend. Therefore, he is phoned many times until he comes for an appointment, after a weekend of consumption. He had remained locked in his room with a knife for 24 hours, convinced that there was a man outside who wanted to hurt him.

Several aspects stand out as part of his psychobiographical history, including a diagnosis of attention deficit hyperactivity disorder (ADHD) at the age of 12, with poor course, early substance abuse in adolescence, and antisocial behavior. His lifestyle from late adolescence has been characterized by nightlife leisure, sexual promiscuity, and drug use.

With regard to drug abuse, he refers to intranasal consumption of cocaine for the last 8 years, in high doses (5-6 grams per weekend) at recreational settings, frequently together with alcohol. Occasionally, he has also consumed diverse substances such as ketamine, GHB, MDMA, methamphetamine, or poppers, depending on availability. When he starts IV mephedrone consumption, he stops the regular use of other substances.

Concerning his medical history, at the age of 18, he is diagnosed with HIV, and two years later with HCV, which is treated for 6 months with peginterferon and ribavirin, showing a sustained virological response. In the following two years, the patient presents two syphilitic infections and two genital candidiases. Three months before admission to the psychiatric unit, he is again infected with HCV.

His mephedrone IV use takes place in private parties, held almost every weekend, where about 4–6 people participate, all men. Parties last 3 to 4 days and participants have continuous sex, with multiple partners and often without using any protection. Consumption amounts to about 3-4 grams per weekend, with redosing almost every hour. Each dose contains about 0.1–0.2 grams of mephedrone, diluted in physiological serum and directly injected intravenously.

After 3 months of maintaining mephedrone consumption, the patient presents delusional paranoid ideation with an intense emotional and behavioral impact. He also develops visual hallucinations involving human forms and cellphone lights, which he believes attempt to record him. During this period, he reports to the police being persecuted twice. At last, he is treated in the emergency room after attempting suicide by drug intoxication.

Upon admission, he is anxious and exhibits distrust and psychomotor restlessness. He also acknowledges feeling physically exhausted and emotionally distressed. The patient denies current craving but admits that craving can occur at any time and with great intensity. He feels that he is being controlled and monitored and is suspicious of his own relatives. He also presents cenesthopathy after the last consumption, which manifests as insects crawling under the skin.

Electrocardiogram, Cranial Magnetic Resonance Imaging, and a blood test are performed, and no irregularities are found. Urine screening for common drugs of abuse came as negative. HBV and syphilis screenings were negative, HIV and HCV positive. The patient started a treatment program with paliperidone (up to 6 mg/day), zonisamide (up to 300 mg/day) for impulsive behavior, and 75 mg/day of pregabalin as an anxiolytic. The psychotic symptoms gradually begin to improve and disappeared after 4 weeks of admission, when he is discharged from hospital.

## 3. Discussion

This case presentation combines diverse interesting aspects to be considered, such as the practice of* slamming* mephedrone in* chemsex parties*, with serious health consequences, mainly severe drug addiction and acute psychotic symptomatology which required the admission to the psychiatric unit. Besides, this practice led to HCV reinfection.

Mephedrone and other SCs acutely increase dopamine and extracellular norepinephrine and serotonin levels because they inhibit their reuptake [16]. They also increase the presynaptic release of monoamines by reversing the normal flow of transporters, in a similar way to amphetamine [6, 17].

The usual presentation of mephedrone is in powder, crystals, tablets, or capsules. The routes of administration are diverse: intranasal, oral, rectal, intravenous, intramuscular, or subcutaneous. The intranasal insufflation is the most common but causes significant irritation. The IV route, on the other hand, has been found to cause an acute burning sensation, complications in the venous system, and skin lesions [18].

The usual doses of mephedrone range between 100 and 200 mg per hour, with higher doses taken orally and smaller ones intranasally [19]. Effects usually begin 30–45 minutes following the first consumption and last between 2 and 5 hours in the case of oral consumption. In the case of intranasal administration, the effects of this substance begin after 10–20 minutes and extend between 1 and 2 hours. When administered intravenously, the effect starts after 10 minutes or less and is maintained for about thirty minutes [20].

The addictive potential of diverse SCs has been shown in animal studies. This is not surprising, given that their mechanism of action resembles that of amphetamine [21]. In humans, preliminary data from a recent study with healthy volunteers comparing the potential for mephedrone abuse with that of MDMA described a more rapid onset and dissipation of effects in the case of mephedrone, leading to a more compulsive pattern of use [22]. Moreover, nearly half of mephedrone consumers surveyed in schools and universities in the United Kingdom, before its prohibition, described this substance as addictive, and 17.5% admitted suffering symptoms of addiction or dependence [23].

Many physical and mental adverse effects related to SCs consumption have been described. Cardiac disorders (tachycardia and hypertension), neurological symptoms (hyperthermia and insomnia), and psychiatric symptoms (agitation, confusion, and suicide ideation) constitute the most common side effects among patients presenting with cathinone intoxication in hospital centres [9, 24]. Also, the deadly potential of mephedrone has been widely cited in the scientific literature [25]. Deaths often occur during the weekends and the following days, as expected given the pattern of consumption of recreational substances [9]. Victims are young people, usually men, with a history of polydrug use [26]. The causes of these deaths are varied, from self-harm or suicide to heart or multiple organ failure [6].

In reference to psychiatric symptomatology, the emergence of brief psychotic symptoms and posttraumatic amnesia are very common [12]. Published cases describe induced psychosis requiring hospitalization and treatment, both in an acute way and after a chronic consumption [27, 28]. In one of the cases, a pattern of abuse of mephedrone following oral, intranasal, and intrarectal administration was described, which induced complex hallucinatory and delusional symptoms, as well as dependence and a marked withdrawal syndrome [29]. Another case relates the possible triggering of schizophrenic-type psychosis [30]. Depending on the administration route, the psychotic symptoms seem to be more intense. In a study where eleven users of IV mephedrone were interviewed, all reported intense paranoia, with two of them showing significant aggressiveness and violent behavior [31]. In the case we present, the critical point of psychotic symptoms occurs during consumption and as delusional paranoid ideation that persists in a sustained way and requires antipsychotic medication during four weeks before symptom remission, requiring psychiatric admission.

Even though epidemiological studies in the general population suggest that in most countries the use of SCs shows a prevalence of 1 to 2% [32], there are some groups of users where mephedrone consumption is more widespread: on the one hand, nightclubbers and, on the other hand, high risk drug users [33, 34]. Regarding the first, in a 2010 survey of regular gay nightclubbers in South London, 54% of the respondents admitted having used mephedrone at least once. The survey was repeated in 2011, and 41% of respondents reported having consumed it that day or having the intention to consume it shortly after [35].

Regarding high risk drug users, consumption of IV cathinones has been described in several European countries. In Hungary and Romania, especially, this practice is much more common, as it has been shown in large cohorts of studied patients [33]. The explanation for these findings has to do with the limited availability of heroin in these countries in 2010-2011 and the ease of obtaining “legal highs” in both the streets and online stores [34]. In fact, some studies based on surveys of the Hungarian population have reported a greater risk of addiction as well as medical and psychiatric complications among users of IV mephedrone in relation to individuals who consume mephedrone orally [36].

Another group exhibiting a growing trend of IV cathinones consumption is MSM who engage in the practice known as* slamming* [37]. Here, mephedrone is usually consumed alone or in combination with other drugs such as *γ*-hydroxybutyrate (GHB), *γ*-butyrolactone (GBL), crystallized methamphetamine, cocaine, and sildenafil to induce disinhibition and enhance sexual experiences. The* chemsex* parties can last from 8 hours to several days and participants usually engage in risky sexual practices with multiple partners, sometimes without protection and exchanging syringes, with a consequent increase in the risk of contracting STDs or viral infections [14, 38]. In fact, a study done in London in a drug addiction clinic for the gay community showed that 75% of users consumed mephedrone only to enhance sex practices and, of these, 80% used IV mephedrone. Of these users, 75% were HIV positive and 70% shared needles [33].

The short duration of IV mephedrone action causes the compulsive repetition of injections to maintain and prolong the effects. Besides this compulsive use, other addictive elements such as craving, uncontrolled binging behaviors, and withdrawal symptoms have been reported [18]. Moreover, the psychoactive and sexual effects of mephedrone can lead to loss of control and risk-taking behaviors, such as unprotected sex, promiscuity, or sharing needles. Our patient shows an absolute lack of control and compulsive consumption of IV mephedrone with intense craving, as well as HCV reinfection.

The practice of* slamming* has been described in London and some French cities [39], although it might become common in other cities. In fact, in Spain, several press articles have been published during the last months, warning about the* chemsex* phenomenon in major cities [40]. It has been described that IV use of mephedrone predominates, along with methamphetamine (less used because of its high price). These parties are publicized in social networks, where people find their coslamming partners and dealers offer the substance [41]. However, psychopathological complications of this practice are just recently being evidenced, because mephedrone* slammers* are beginning to seek drug related treatments or are being referred to emergency rooms as a consequence of mephedrone physical or psychiatric side effects.

As far as we know, this case is the first one describing a psychotic outbreak induced by* slamming* mephedrone in* chemsex parties*, in Spain. This report raises concern, as the consumption of mephedrone and other SCs is becoming more frequent in sex related practices, despite the consequent mental and physical health risks.
